# Initial toxicity assessment of ICON6: a randomised trial of cediranib plus chemotherapy in platinum-sensitive relapsed ovarian cancer

**DOI:** 10.1038/bjc.2011.334

**Published:** 2011-08-30

**Authors:** F A Raja, C L Griffin, W Qian, H Hirte, M K Parmar, A M Swart, J A Ledermann

**Affiliations:** 1 Cancer Research UK and UCL Cancer Trials Centre, UCL Cancer Institute, University College London, 90 Tottenham Court Road, London W1T 4TJ, UK; 2 Medical Research Council, Cancer Trials Unit, London, UK; 3 Juravinski Cancer Centre, Hamilton Health Sciences, Hamilton, Ontario, Canada

**Keywords:** ICON6, relapsed ovarian cancer, cediranib

## Abstract

**Background:**

Cediranib is a potent oral vascular endothelial growth factor (VEGF) signalling inhibitor with activity against all three VEGF receptors. The International Collaboration for Ovarian Neoplasia 6 (ICON6) trial was initiated based on evidence of single-agent activity in ovarian cancer with acceptable toxicity.

**Methods:**

The ICON6 trial is a 3-arm, 3-stage, double-blind, placebo-controlled randomised trial in first relapse of platinum-sensitive ovarian cancer. Patients are randomised (2 : 3 : 3) to receive six cycles of carboplatin (AUC5/6) plus paclitaxel (175 mg m^−2^) with either placebo (reference), cediranib 20 mg per day, followed by placebo (concurrent), or cediranib 20 mg per day, followed by cediranib (concurrent plus maintenance). Cediranib or placebo was continued for 18 months or until disease progression. The primary outcome measure for stage I was safety, and the blinded results are presented here.

**Results:**

Sixty patients were included in the stage I analysis. A total of 53 patients had received three cycles of chemotherapy and 42 patients had completed six cycles. In all, 19 out of 60 patients discontinued cediranib or placebo during chemotherapy because of adverse events/intercurrent illness (*n*=9); disease progression (*n*=1); death (*n*=3); patient decision (*n*=1); administrative reasons (*n*=1); and multiple reasons (*n*=4). Grade 3 and 4 toxicity was experienced by 30 (50%) and 3 (5%) patients, respectively. No gastrointestinal perforations were observed.

**Conclusion:**

The addition of cediranib to platinum-based chemotherapy is sufficiently well tolerated to expand the ICON6 trial and progress to stage II.

Ovarian cancer is the leading cause of death from gynaecological malignancy, with over 230 000 women diagnosed and 140 000 women dying per year. Although many women respond well to primary therapy with surgery and chemotherapy, the majority will relapse and die from their disease. Better treatments for first-line therapy and recurrent disease are urgently required. Current standard treatment for women relapsing 6 or more months after completion of first-line therapy is platinum-based chemotherapy, usually carboplatin in combination with paclitaxel (Parmar *et al*, 2003), gemcitabine (Pfisterer *et al*, 2006) or liposomal doxorubicin (Pujade-Lauraine *et al*, 2010)

Inhibition of angiogenesis is emerging as an important strategy in the treatment of cancer. Vascular endothelial growth factor (VEGF) is a key mediator in this process, and a number of drugs have been developed to target VEGF and its associated receptors. Cediranib is an oral, small-molecule, VEGF receptor tyrosine kinase inhibitor. It is a potent inhibitor of all three VEGF receptors (VEGFR 1, 2 and 3) and c-Kit. Cediranib has demonstrated broad antitumour activity as monotherapy and in combination with certain chemotherapy regimens. Two phase II studies demonstrated encouraging activity of cediranib monotherapy, with response rates of 17–23% even in heavily pretreated ovarian cancer patients (Hirte *et al*, 2008; Matulonis *et al*, 2009).The initial dose of single-agent cediranib was 45 mg, which was subsequently reduced to 30 mg after encountering toxicity in the first group of patients. The most frequently observed toxicities were grade 3 hypertension (25–46% of women), grade 3 fatigue (17–24%) and grade 3 diarrhoea (13%). Other toxicities noted include nausea and vomiting, proteinuria, muscle weakness, haemorrhage, dysphonia, derangement of transaminases and thrombosis. Gastrointestinal perforation has been observed in patients receiving cediranib, although the incidence appears to be lower than for bevacizumab (Schmoll *et al*, 2010).

Evidence of activity and manageable toxicity provided the basis for investigating cediranib in the International Collaboration for Ovarian Neoplasia 6 (ICON6) study. The ICON6 trial is a double-blind, placebo-controlled randomised trial in women with ‘platinum-sensitive’ ovarian cancer in first relapse. It is evaluating the addition of cediranib to platinum-based chemotherapy, concurrently during chemotherapy and continued as a maintenance therapy for 18 months (Figure 1). The ICON6 trial is a three-stage academic trial developed by the Gynecologic Cancer Intergroup (GCIG), led by the Medical Research Council, UK, funded by Cancer Research UK and partially supported by AstraZeneca. It has a novel multi-stage, multi-arm design that allows a number of questions to be answered in a seamless fashion: is the addition of cediranib to chemotherapy safe and beneficial, and does maintenance treatment with cediranib following chemotherapy confer an additional advantage?

The primary aim for stage I was to determine the safety and feasibility of adding cediranib to platinum-based chemotherapy. If concurrent cediranib and chemotherapy were found to be tolerable at the stage I analysis, then the trial would be expanded and proceed to stage II. The primary outcome measures for stage II is activity as assessed by effect on progression-free survival (PFS), and for stage III it is overall survival (OS). The aim is to recruit 2000 patients for the third-stage analysis.

The International Collaboration for Ovarian Neoplasia 6 trial was first opened to recruitment in December 2007. Soon after, emerging safety data from other trials indicated that the combination of 30 mg of cediranib with chemotherapy was associated with significant toxicity, although the activity of the combination was promising (Goss *et al*, 2010). An excess of toxicity and problems with compliance were also observed in ICON6. These data led AstraZeneca to recommend a reduced dose of cediranib of 20 mg daily when given in combination with chemotherapy. Thus, the dose of cediranib in ICON6 was reduced to 20 mg daily after 30 patients had been randomised at the higher dose. Here we report the results from the blinded, stage I safety analysis of the ICON6 trial.

## Patients and methods

Eligibility criteria consisted of patients with histologically confirmed epithelial ovarian cancer, primary peritoneal or fallopian tube cancer, with recurrent disease (measurable or non-measurable) seen on CT or MRI more than 6 months after the last cycle of the first-line chemotherapy. Patients were eligible regardless of the type of the first-line chemotherapy. An ECOG performance status of 0 or 1 and adequate organ function were required.

### Randomisation, stratification and treatment

Patients are randomised in a 2 : 3 : 3 ratio to one of three treatment arms after stratification for: GCIG group, first-line chemotherapy (paclitaxel *vs* no paclitaxel), duration of relapse free interval (6–12 months *vs* >12 months), planned chemotherapy regimen (carboplatin/cisplatin *vs* carboplatin/cisplatin+paclitaxel) and any previous bevacizumab treatment (yes or no). An unequal ratio of randomisation is being used to adequately power the comparison of the two cediranib arms (B+C).

The dose of carboplatin (infused over 30–60 min) was AUC 5 (GFR measured) or AUC 6 (calculated dose), and for paclitaxel was 175 mg m^−2^ (infused over 3 h). Where cisplatin was used, the recommended dose was 75 mg m^−2^. Chemotherapy cycles were planned to be administered every 3 weeks. All toxicities were graded according to CTCAE v 3.0. Protocol-defined dose reductions of chemotherapy were performed if necessary.

### Dose modifications

Patients discontinued trial drug permanently if they developed gastrointestinal perforation, arterial thromboembolic events (e.g., myocardial infarction, cerebrovascular accident or transient ischaemic attack), reversible posterior leucoencephalopathy syndrome or grade 4 toxicity secondary to trial drug (e.g., hypertension, proteinuria or haemorrhagic event). The trial drug was reduced to 15 mg if patients developed any grade 3 toxicity secondary to the trial drug. A delay in treatment for longer than 2 weeks due to toxicity resulted in permanent discontinuation of trial drug.

At the start of the trial, relatively little was known about the safety of cediranib in combination with platinum-based chemotherapy in ovarian cancer. Stage I was therefore restricted to a small number of selected sites, in the United Kingdom and Canada, experienced in management of patients with advanced ovarian cancer and early phase clinical trials. Clinical guidance for the management of known common dose-limiting toxicities of hypertension, diarrhoea, fatigue and proteinuria were developed. These guidelines were provided for use by clinicians and nurses alongside the clinical protocol, with detailed steps on how to manage common toxicities. In general, short dose interruptions (i.e., 2–5 days) of trial drug were recommended for the management of adverse events. Once symptoms had resolved to CTCAE grade 1 with supportive care, the trial drug could be restarted. For management of hypertension, patients were provided with a blood pressure monitor for home monitoring and instructed to call their primary care or hospital physician if the self-checked BP exceeded 140/90 mm Hg. A detailed algorithm for initiation of appropriate antihypertensive treatment was provided for management of hypertension.

### Evaluation

Patients were seen on day 1 of each cycle of chemotherapy, and then they attended a week 21 visit at which point patients on arm B (concurrent cediranib) were switched to placebo. Daily blood pressure readings were taken for the first two cycles and at the beginning of each cycle thereafter. Patients who completed at least four cycles of chemotherapy were eligible to continue study drug, even if they had stopped chemotherapy for toxicity or patient choice (not for disease progression). Following completion of chemotherapy, patients attended safety follow-up visit every 6 weeks while on study drug for up to 75 weeks and then 6 weeks after finishing the study drug.

Progression was determined by CT or MRI of abdomen and pelvis and not by CA 125 values, although levels were taken at baseline, at each cycle of chemotherapy and then every 6 weeks while on trial drug treatment. The CT or MRI scans were performed at baseline, after 6 cycles of chemotherapy and at 12 and 18 months from randomisation for all patients who had not progressed. Additional scans could be performed at any point if clinically indicated.

### Statistical methods

The primary end point of stage I was safety. At the start of the trial, the first-stage analysis was planned when ∼33 patients in total in the two cediranib arms (B+C) had completed three cycles of chemotherapy. The rationale for this was that this was a reasonable number of patients for assessment of toxicity. Although all toxicities were to be considered, particular consideration would be given to CTCAE grade 3 and 4 toxicities associated with cediranib (i.e., severe hypertension, grade 3 fatigue and diarrhoea) or events that required dose reduction or discontinuation of trial drug. As a guideline, if there was good evidence that the grade 3 and 4 adverse event rate exceeded 15% of patients for any of these events (or other grade 3 or 4 toxicities identified), then the nature of the research arms of the trial would be reconsidered. This would occur if more than 10 of the 33 patients on trial drug experienced these events (in which case the lower end of the 95% confidence interval would exceed 15%).

However, chemotherapy alone produces a significant proportion of grade 3 and 4 toxicities, and it is not always clear whether toxicity is solely due to trial drug. After discussion between the Trial Management Group, Trial Steering Committee and Independent Data Monitoring Committee (IDMC) the stage I guidance was modified so that if any grade 3 or 4 toxicities (excluding alopecia) in the experimental arms were 15% higher than the control arm, then the nature of the research arms of the trial would be reconsidered (i.e., lower limit of the 95% confidence interval of the difference was more than 15%).

At the IDMC meeting in November 2008, it was recommended that the dose of the trial drug should be reduced to 20 mg and the stage I analysis should be performed when at least 50 patients had been randomised and received a minimum of three cycles of chemotherapy with 20 mg dose of trial drug. The data set was locked for this analysis on 18 November 2009.

## Results

The results presented are of the blinded ICON6 stage I safety analysis. The toxicities of the reference arm and experimental arms are not presented separately, as this would compromise the integrity of the blinded study. The trial is ongoing, with unblinded results reviewed only by the IDMC.

### Patient characteristics

Between December 2007 and November 2009, 108 patients were randomised. Of these, 78 patients were randomised to a starting dose of 20 mg per placebo. Baseline patient characteristics are summarised in Table 1.

The median age was 62 years (range 32–82). The majority (83%) had platinum plus paclitaxel as the first-line therapy, and 94% was planned to receive platinum plus paclitaxel as part of the second-line treatment. The treatment-free interval from completion of the first-line chemotherapy and randomisation was 6–12 months in 44% of patients and >12 months in 56% of patients. In all, 93% had not received any previous bevacizumab.

### Treatment

A total of 53 out of 60 patients randomised to 20 mg per placebo dose had received three cycles of chemotherapy, and 49 patients had been in the study long enough to have received six cycles of chemotherapy. In all, 42 of the 49 (86%) patients had completed six cycles. Three patients had fewer than six cycles because of adverse events or intercurrent illness, three patients died before completing chemotherapy and the reason for one patient not receiving six cycles of chemotherapy was not reported.

### Safety

The stage I safety analysis population was predefined in the protocol. Sixty patients taking cediranib 20 mg per placebo met these criteria. All grade 3 and 4 toxicities experienced are given in Table 2. A total of 33 (55%) patients experienced grade 3 or 4 toxicity during chemotherapy. In all, 13 patients (22%) experienced grade 2 hypertension. Nineteen patients discontinued trial drug during chemotherapy; nine stopped owing to an adverse event or intercurrent illness; one owing to disease progression; one owing to patient choice; one owing to administrative reasons; and four owing to other reasons (a combination of adverse events and patient choice). There were three deaths (bowel obstruction, ovarian cancer and congestive cardiac failure).

Trial drug was administered without a dose reduction to 31 out of 60 patients during the first three cycles of chemotherapy; of these 14 patients omitted at least one tablet of the trial drug. Thirteen patients had a dose reduction of study drug to 15 mg; 10 of these patients continued with chemotherapy and trial drug, whereas three subsequently stopped trial drug but continued with chemotherapy. Ten patients stopped trial drug without a dose reduction but continued on with chemotherapy, and six patients had chemotherapy and trial drug stopped simultaneously.

## Discussion

The stage I analysis of ICON6 has demonstrated that it is feasible to add cediranib to carboplatin/cisplatin and paclitaxel chemotherapy without major unexpected toxicities. In all, 86% of patients in the safety population completed six cycles of chemotherapy. This compares favourably to the ICON4 study of relapsed ovarian cancer (Parmar *et al*, 2003) in which 72% of patients completed six cycles. Sixty-nine percent of patients had trial drug for the first three cycles of chemotherapy: 52% had received cediranib/placebo 20 mg per day and 17% needed to have cediranib/placebo dose reduced to 15 mg per day as part of the toxicity management.

The reported adverse events have so far been manageable with dose reductions, short ‘study drug holidays’ and use of detailed clinical guidelines for management of common toxicities. In addition, patients were carefully briefed on possible side effects, monitored closely and provided with easy access to healthcare professionals. Early recognition of side effects and prompt intervention were heavily emphasised to investigators to minimise symptoms and improve tolerability of cediranib. Without these measures, it is likely that there would have been greater toxicity observed.

However, it is possible that we have underestimated the toxicity of cediranib in combination with chemotherapy, as the number of patients in the safety stage was small and included the control arm that did not contain cediranib. In addition, some patients had not completed six cycles of chemotherapy. Thus, cumulative toxicities and late effects may not yet have developed. However, the frequency of toxicities in the unblinded arms reviewed by the IDMC was below the ‘cutoff’ for stopping the trial.

Cediranib has been tested in a broad range of advanced cancers, such as non-small-cell lung cancer (NSCLC), colorectal cancer and glioblastoma. The BR24 trial in first-line advanced NSCLC investigated the addition of cediranib to carboplatin and paclitaxel (Goss *et al*, 2010). In the primary analysis, an improved response rate and PFS were observed, but with an imbalance of toxicity in the cediranib arm compared with the control arm. Patients in the cediranib 30 mg arm had higher incidences of hypertension, hypothyroidism, hand–foot syndrome and GI toxicity compared with placebo. The trial did not meet its predefined end point and did not proceed to phase 3. However, based on the encouraging efficacy data, a second study (BR29 trial) was launched in advanced NSCLC adding placebo or cediranib 20 mg in combination with carboplatin plus paclitaxel. This trial has now completed recruitment, and presentation of the results is awaited. The HORIZON III phase 3 study in colorectal cancer compared FOLFOX 6 with cediranib 20 mg or bevacizumab. No statistically significant difference was observed in PFS, OS or ORR; however, the predefined boundary for non-inferiority was not met. With these data, and that from the HORIZON II study, AstraZeneca announced that they were not seeking a license for cediranib in colorectal cancer (Schmoll *et al*, 2010). In glioblastoma, initial promise in a phase 2 trial led to the development of the phase 3 REGAL study in which patients with recurrent glioblastoma were randomised to cediranib 30 mg, cediranib 20 mg plus lomustine or lomustine alone. However, neither cediranib arm demonstrated a significant advantage over lomustine alone for PFS, OS or RR (Batchelor *et al*, 2010).

Although the major phase 3 trials with cediranib have produced disappointing results, promising activity has been seen with cediranib monotherapy in ovarian cancer (Hirte *et al*, 2008; Matulonis *et al*, 2009). Furthermore, the recently presented OCEANS trial (Aghajanian *et al*, 2011), demonstrating a significant improvement in PFS when bevacizumab is added to carboplatin and gemcitabine, has provided ‘proof of principle’ for the use of antiangiogenic agents in recurrent ovarian cancer. International Collaboration for Ovarian Neoplasia 6 remains a crucial study in evaluating the role of an oral antiangiogenic tyrosine kinase inhibitor in recurrent ovarian cancer.

The study is now in stage II, with expansion of sites in the United Kingdom and Canada and introduction of sites in Australia, New Zealand, Korea and continental Europe. Recruitment of patients into the trial has accelerated. The protocol has been modified to allow the continuation of trial drug beyond 18 months until progression if the patient appears to be benefiting. In addition, gemcitabine and carboplatin may be used as an alternative to carboplatin and paclitaxel. Although stage II is in progress, toxicity of the gemcitabine/platinum combination will be assessed by the IDMC after 30 patients have received this combination with cediranib. The combination of liposomal doxorubicin and carboplatin was not permitted despite the favourable results of the CALYPSO trial (Pujade-Lauraine *et al*, 2010) because of the possibility of cardiac toxicity observed in patients treated with cediranib plus doxorubicin (Denduluri *et al*, 2007). Following the publication of the HORIZON and REGAL trials, an early interim analysis of stage II is planned for evaluating activity as assessed by effect on PFS. On the basis of these results, a decision will be made about continuing to stage III in which OS will be the primary end point.

The design of the trial allows a number of questions to be answered in a sequential fashion. The migration of the trial through three stages without cessation of randomisation produces a time-efficient and cost-effective approach using data on all the patients randomised. Stage I has primarily confirmed the safety of carboplatin and paclitaxel in combination with cediranib.

## Figures and Tables

**Figure 1:**
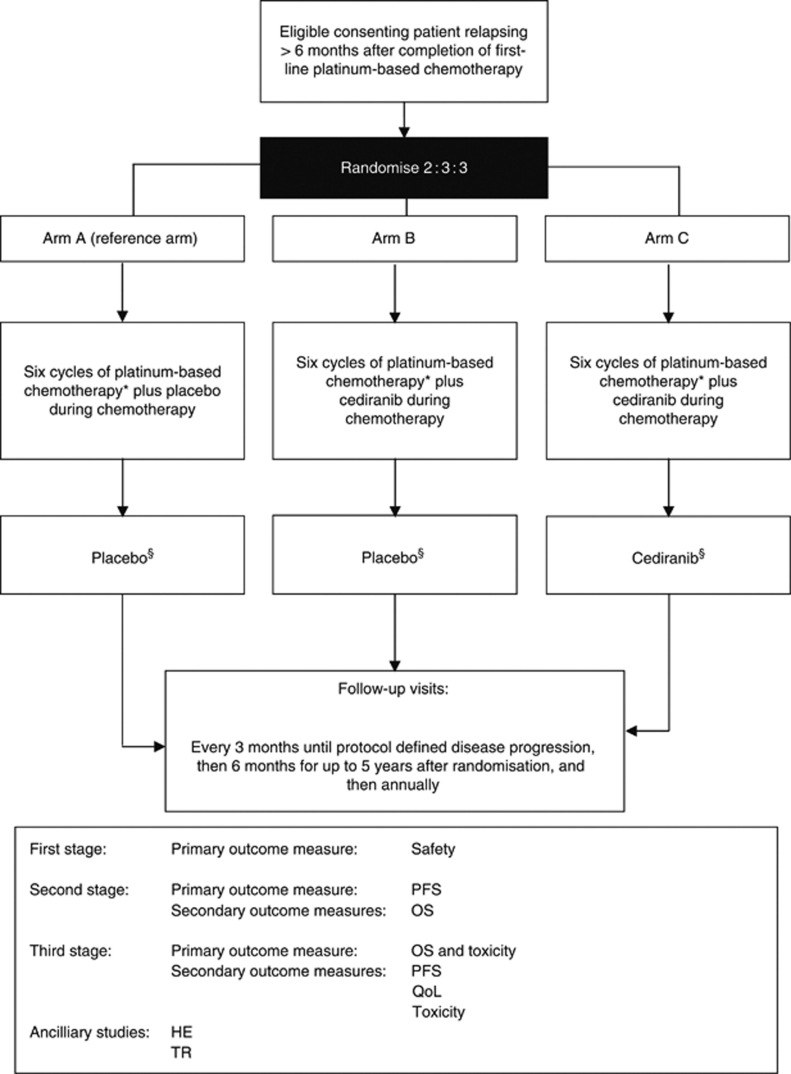
International Collaboration for Ovarian Neoplasia 6 trial schema. ^*^The recommended chemotherapy in ICON6 is carboplatin plus paclitaxel; however, treatment with other platinum-based chemotherapy regimens may be permitted, that is, single-agent carboplatin or cisplatin or cisplatin in combination with paclitaxel. ^§^Trial drug (placebo or cediranib) continues for 18 months. HE=health economics; OS=overall survival; PFS=progression-free survival; QoL=quality of life; TR=translational research.

**Table 1:**
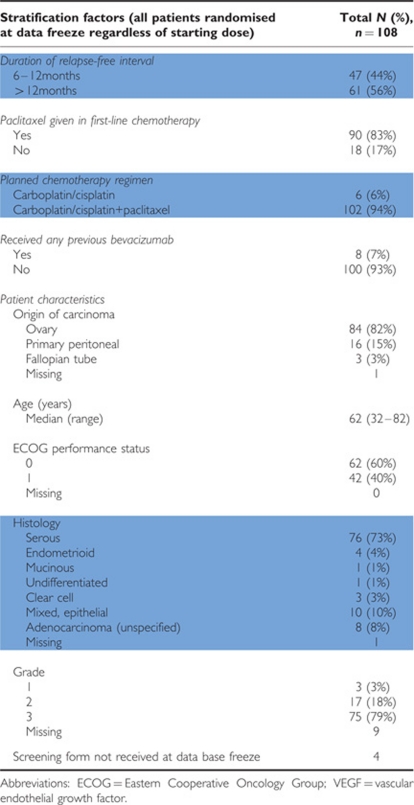
Baseline characteristics

**Table 2:**
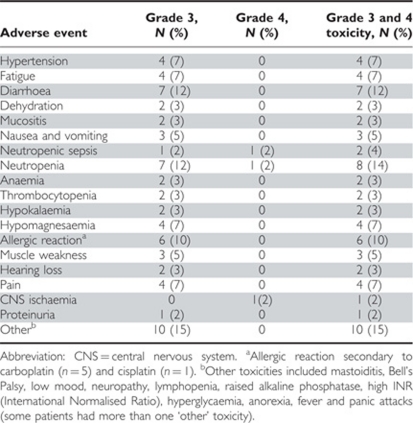
Grade 3/4 toxicity experienced by stage 1 safety analysis population (*n*=60)
